# Topics of Public Interest

**Published:** 1917-02

**Authors:** 


### TOPICS OF PUBLIC INTEREST

---

**Deaths of Physicians.** The Journal of the A. M. A. published 2.196 death notices in 1916, 14.08: 1000 of an estimate of 158,000 American (including Canadian) physicians. The average, 1902-16, was 15.61. Probably the actual death rate is higher as many physicians drop from professional acquaintance before their death. The ages ranged from 23 to 99, with an average of 25 days less than 60. Years of practice ranged from the first to 77, averaging over 32. 596 were fellows of the A. M. A. The principal causes were senility, 337; heart disease 215; pneumonia 208; cerebral haemorrhage 204; accidental 112; operations 98; nephritis 72; tuberculosis 62; carcinoma 49; appendicitis 40; suicide 39; homicide 15, etc. Of the 112 deaths due to accident, 28 were ascribed to automobiles alone and 17 to grade crossing collisions between trains and automobiles, the total being 45, a trifle over the 2% estimate in the editor's article in the Jan. issue, of this journal.

---

**Radiator Solutions.** The latter part of the winter is the dangerous time, owing to the greater tendency of alcohol to evaporate so that the solution, though nominally strong enough to resist the minimum temperature, is considerably below this strength. For example, a solution supposedly of 15% strength, was found to be of less than 10%. As an approximate rule, every 10% of alcohol lowers the freezing point 14° F. below 32°. Also, as an approximate rule, if the radiator is replenished with a solution 5% higher than what is intended, the greater volatility of alcohol will be compensated. Except in an emergency, strong or dilute alcohol should be added only when the engine is cold and just before running. Otherwise, the alcohol will rapidly evaporate and the contents of the radiating system will not mix so as to prevent freezing. A good practical test of contents is to withdraw a little into a bottle—for instance in the evening or, at any rate, after the engine has run long enough to insure thorough mixing—either by siphoning from the top or through the pet cock at the bottom and expose to cold. Usually alcohol insufficient to prevent freezing will at least allow freezing in a slushy, non-expansive form. The freezing point can be quite accurately determined by immersing a thermometer and noting the point at which nearly complete melting takes place.

---

**Tethelin** is the name applied to a pituitary extract, isolated by Dr. T. D. Robertson of the University of California. It is said that experiments tend to support the claim that stature may be quite definitely controlled by the administration of tethelin or the suppression of the secreting function. It is scarcely necessary to state that the somewhat sensational notices in the daily press should not be accepted at their face value.

---

**Professional Opposition to Prohibition.** Drs. Wm. H. Welch, W. S. Halstead, Hugh H. Young and Julius Friedenwald, have signed a public protest against proposed legislation in Maryland, which would prevent the use of alcohol in any form in medicines in laboratories, hospitals and medical practice, unless allowed by subsequent legislation. There is also proposed, national legislation, to prevent the mailing into a prohibition state of a periodical advertising an alcoholic liquor. It is obvious that, except from a very extreme standpoint, the cause of temperance is best served by recognizing that alcohol is a drug, and except for its use in the arts and commercially, nothing but a drug. This implies that alcoholic liquors should be advertised in medical journals—and when ideal conditions are reached only in medical journals—and due allowance for this fact should be made in all legislation.

---

**The New York State Civil Service Commission** announces examinations on Feb. 24 to fill various positions. Those more or less of interest to medical readers are: Veterinarian, Dept. of Agriculture, $7-10 a day or $1200-2500 per year; Muscle Tester (Woman 21-45 years) for after care of Infantile Paralysis, Dept. of Health, $75 a month; Supervising nurse (same requirements) $100 a month; Assistant Inspector of Physical Training, Military Training Commission, $2500-3000. No blanks will be sent out after Feb. 13 and none will be received after Feb. 15.

---

**A Tentative Draft of an Act for Health Insurance** has been published by the Am. Assn. for Labor Legislation in N. Y. City. This is stated to be approved by the Council of the Medical Society of the State of N. Y. It provides for a state medical advisory board, elected by the State Medical Societies instead of a single physician member of the state commission. (Note that the definition of "State Medical Societies" should be carefully considered). Medical representation on local boards is also provided for and there is proposed a fair representation of health department, general practitioners and specialists. In case of dispute, reference is to be made to an arbitration committee composed of representatives of the medical profession, of the local health insurance fund and an impartial chairman. Certificates of disability are to be issued only by medical officers not permitted to practice, and only on recommendation by the attending physician.

**The University of Toronto** graduated on Dec. 11, a class of 47, all of whom have enlisted for war service. The senior class has been working without vacation in order to graduate as early as possible.

**Patent Bread,** unfermented and apparently what was later and is still known in London as aerated bread is mentioned in our issue of June 1862 as just obtainable in Buffalo, being made by Mr. Fuller. This was prepared by forcing carbon dioxid through the dough instead of developing this gas by yeast.

**Stature.** 1 man in 208 is said to exceed 6 feet.

**State Department of Health.** Division of Communicable Diseases. Deaths in New York State 1915—Diphtheria 1754; Typhoid Fever 750; Measles 834; Whooping Cough 749; Scarlet Fever 409.

The reports from Erie county during last month indicate that diphtheria was unusually prevalent. The Department, therefore, offers the following suggestions regarding the control of this disease:

1. Take cultures from all suspicious cases of diphtheria and isolate them until a negative report is received. The health officer has a supply of cultures and antitoxin for free distribution.

2. As soon as the diagnosis is made report at once to the health officer and make out a list of persons in contact with the case.

3. Take cultures from persons who have been exposed to the patient. Those from whom negative reports are obtained need not be isolated, but if isolation will be violated the whole household must be quarantined. The health officer will decide on this matter.

4. The discharges from the nose and throat should be wiped away with cloths. The soiled cloths should be placed immediately in a basin containing 5% carbolic acid or placed in a paper bag and later burned.

5. If the case is on a dairy farm, see that Regulation 37, Chapter II of the Sanitary Code is observed.

6. If the case is clinical diphtheria, give antitoxin before waiting for a laboratory report.

7. There is rarely a death when antitoxin is given during the first day of the disease. If the patient is very sick the intravenous injection of antitoxin will give a quicker response. Further details of method for administering antitoxin will be found on the wrapper of container.

8. Examine exposed infants frequently and give antitoxin when evidence of illness first appears.

9. The Schick test will detect those who are immune.

10. A permanent immunity can apparently be given those who are not immune. See Jour. Am. Med. Assoc. Dec. 25, 1915. Park & Zinger.

HERMANN M. BIGGS, Commissioner of Health.

---

**Quack Advertisements.** Assemblyman Robert M. Marsh has introduced a bill making it a misdemeanor to publish advertisements of quacks or so-called specialists. (N. Y. State).

---

**Bread Bill.** Senator James W. Walker has introduced a bill requiring that bread must be labeled with a statement of all ingredients aside from the usual ones (flour, water, salt and yeast) and prohibiting the use of poisonous or injurious ingredients. This bill was defeated last year, largely through the influence of the Ward Baking Co. We have no disposition to oppose this Co., though its vehicles do seem to obstruct traffic more than any other single factor, but the bill on its face seems a good one.

---

**Liquor and Tobacco Statistics.** In spite of the increase of prohibition states, last year's consumption of whisky reached the record mark of 146 1/2 million gallons for the U. S., as compared with 124 1/2 for 1915. Beer slightly exceeded 61 million barrels, an increase of over 2 million as compared with 1915 but decidedly below the consumption for several preceding years. The seasonal fluctuation in the consumption of liquors is interesting. 80½ more whisky is used in Nov. and Dec. than in the summer while 50% more beer is used in the summer than in the winter. Over 25 billion cigarettes were produced in 1916, as against not quite 18 billion in 1915. The former is the highest record reached. It is possible that contributions to European soldiers account somewhat for the increase though it is ascribed to the growth of the habit among women. It is possible that a compensatory diminution in other forms of smoking tobacco will be observed when the statistics are compiled. If so, the increase in cigarettes may be considered salutary.

---

**Increase in Fees.** Boston physicians, to meet the H. C. L., have established rates of $3 for visits between 8 and 5; $4

between 5 and 9 p. m.; $5 for night calls from 9 p. m. to 8 a. m.

---

**Bellevue Hospital** has added an ophthalmologic service of a present capacity of 50 beds, in charge of Chas. H. May, assisted by Julius Wolff and John M. Wheeler.

---

**Centenarian.** Mrs. Mary Talbot of Ottumwa, Ia., a former slave, whom we have mentioned before, passed her 120th birthday, Dec. 26, 1916.

---

**California Law Requiring License of Drugless Practitioners Upheld.** The law of California after exempting regular physicians and surgeons, whose education and license are otherwise provided for, establishes certain requirements, including a knowledge of physiology, for osteopaths, neuropaths, optometrists, chiropractors and other drugless healers, and provides for their license. They were enacted in 1913. On Jan. 8, the U. S. Supreme Court dismissed two suits, brought, respectively by a chiropractor and a "drugless ophthalmologist," on the contention that the law was unconstitutional. Christian Scientists and others treating disease solely by prayer, are exempt. (Note: While we have progressed a long way from the old contention that the practice of medicine meant largely the use of drugs, vestiges of this contention remain in various laws attempting to discriminate between regular and irregular practice. It should be made even more plain that at present, to the public and to legislators and courts, that this distinction is impracticable and that the licensing of a practitioner depends to a very minor degree upon his possibility that without proper education, he will do direct harm with drugs. Very few cases of poisoning by drugs given or prescribed by physicians occur and the few cases that do occur are due mostly to drunkenness. It is not even very frequently that poisoning occurs from drugs administered by pharmacists or irregular practitioners. Those ignorant of the proper use and dose of a drug will usually avoid it altogether, or will employ proprietary tablets and mixtures of stated safe dose. Educational requirements and licenses for physicians and their competitors should be clearly understood to be needed, not so much to prevent direct harm from what the practitioner may do as to what he may neglect to do through ignorance. Ed.)

---

**Equipment of Buffalo Municipal Hospital.** $350,000 has been asked by the West Farm Hospital Commission, to equip the hospital now in course of erection on Grider St. $600,000

was appropriated for its construction and the purchase of the site.

---

**A Typhoid Carrier** has been found among the patients at the Gowanda State Hospital for the Insane. This is the probable explanation of several cases reported.

---

**Automobile Deaths.** 729 persons were killed in N. Y. State in 1916, as compared with 663 in 1915. The deaths for N. Y. City were 329. In N. Y. City, trolleys killed 78 and wagons 74. In N. J., the deaths from automobiles were 215 as compared with 241 for 1916. In N. Y. State, only 82 persons were killed at R. R. crossings, as compared with 122 for 1915.

---

**Medical Military Preparedness.** Representatives of most of the medical schools of the U. S., at a meeting called by the Commission for National Defense, practically unanimously agreed to co-operate by establishing special courses in military practice, for the senior classes. It is to be hoped that these courses will be thrown open to the profession generally.

---

**Death of Old Twin.** John Tunstall of Scranton, died Jan. 2, aged 90, survived by his twin brother Stephen. They were believed to be the oldest twins in the country.

---

**Buffalo Climate.** The highest temperature for 1916 was 92° F., July 19. On three other days the temperature reached 90°. Zero weather was reached only on Feb. 1 and Mch 18. For Dec. there was only one clear day. The total precipitation, 3.37 inches and the average temperature, 28°, were merely averages of many years though the Weather Bureau admits that more of the precipitation was in the form of snow than is usual. The principal trouble that we find with the weather bureau, is that, after suffering from heat or cold, or rain and mud, or deep snow, it takes away both the satisfaction of bragging about conditions and the hope of better times in the years to come, by assuring us that we have had just about the average. And, if we look about for a place where the weather is reasonably decent, it is infested with snakes or yellow fever or disagreeable entozoa or the inhabitants are either sanguinary in their customs or, at any rate, not agreeable as companions.

---

**The De Graff Memorial Hospital of N. Tonawanda,** through the Superintendent, Dr. John A. Rafter, reports 626 cases treated in 1916 and 361 operations performed.

**N. Tonawanda** reported 417 births and 201 deaths for 1916, as compared with 420 and 179 for 1915.

---

**Birth and Infantile Death Statistics,** issued by the U. S. Census Bureau apparently support the claims of birth restrictionists. It is evident, however, that while the infantile death rate tends to rise, in a sense proportionately, with the birth rate, the thousands taken as a basis for the respective rates are different. However, it must be admitted that the same factors which tend to cause a high infantile mortality in the first year of life, do, to a large degree, continue not only throughout early childhood but much later and that a high birth rate may eventually not produce a rapidly increasing population. Up to about 50 years ago, an average of 4 births per marriage meant a stationary population. Today probably 3 births per marriage result in 2 survivals to maturity, compensating for the inevitable death of the parents.

| Country. | Birth rate, per 1000 pop. | Infant-mortality rate, per 1000 born alive. |
|---|---|---|
| United States (registration area only: 1915) | 24.9 | 100 |
| England and Wales (1913) | 24.1 | 108 |
| France (1912) | 10.0 | 78 |
| German Empire (1912) | 28.3 | 147 |
| Austria (1912) | 31.3 | 180 |
| Russia in Europe (excluding Finland and the provinces of the Vistula and of the Caucasus: 1909) | 44.0 | 248 |
| Italy (1913) | 31.7 | 137 |
| Spain (1913) | 30.4 | ... |
| Norway (1913) | 25.3 | 65 |
| Sweden (1912) | 23.8 | 71 |
| Denmark (1913) | 25.6 | 94 |
| Belgium (1912) | 22.6 | 120 |
| Holland (1913) | 28.1 | 91 |
| Switzerland (1913) | 23.1 | 96 |
| Japan (1911) | 34.1 | 157 |
| Australia (1913) | 28.3 | 72 |

---

**American Physicians Needed in England.** As England cannot commission foreigners in the army, it is proposed to offer inducements to some hundreds of young American physicians to serve in the insular hospitals and thus release British physicians for military service.

**Cancer Mortality.** The Census Bureau has just issued statistics based on the year 1914. For the registration are which now comprises about 2/3 of the population, the rate has increased from 63 to 79.4:100,000 population, from 1910 to 1914. This may of course be due to greater accuracy in diagnosis, or possibly to inaccuracy due to the propaganda about cancer. In 1910 the rate was 88 for places of over 10,000 population, and only 69.6 for smaller places, in spite of the expectation that the smaller places would have a proportionately larger aged population. Probably the difference is due to failure to discriminate between deaths IN the hospitals of cities and deaths OF residents of the same cities. At any rate this failure, as we have several times pointed out, applies to typhoid statistics. The lowest rate was 45.8 for Utah, the highest 109.9 for Vermont, other wide discrepancies between states being apparently due to differences in average age of population and in the proportionate numbers of negroes and Caucasians. Whether the rate of 80 for the latter and 56.2 for the former is essentially due to racial differences or to the greater vulnerability of the negroes to other diseases and hence average less longevity, is to be questioned. However, the comparison militates against the theory that such diseases as tuberculosis and syphilis, in the individual or by means of establishing a hereditary taint, favor the occurrence of cancer; as well as the theory that hygienic conditions in general or in regard to diet, etc., cause cancer. The rate for females is 96.8, for males 62.4. It is often said that the female has the same general liability to cancer as the male, plus that due to the vulnerability of essentially sexual organs, including the breast. This is an under-statement. About 26% of all cancers are of these organs so that, according to this dictum, females should have just about 1/3 more cancer deaths than males, whereas the liability of females is actually over 50% greater. As we have elsewhere pointed out (Medical Record, April 18, 1914) cancer is not so far as deaths are concerned, a disease of the menopause. On the contrary, the mortality rate keeps on increasing with age, though our figures, based on earlier reports and referred to deaths in the age period instead of to the corresponding number of persons living in the age period, showed a falling off after the 70th year, to a really low proportionate mortality for advanced ages. The present statistics (1914) the rate being to 100,000 living, the population of the corresponding age period are as follows: under 25, 2.8; 25-34, 13.9; 35-39, 42; 40-44, 78.6; 45-49, 128.6; 50-54, 199.7; 55-59, 305.9; 60-64, 393.1; 65-69, 516; 70-74, 672.3; 75-79, 766.6; 80-84, 889.6; 85 and over, 875.6.

The anatomic distribution of cancers is shown in the following table. Note especially that the rectum and anus, if these statistics are correct, furnish by no means the proportionate number of all intestinal cancers usually taught, and that intestinal cancers in general are far more frequent as compared with gastric cancers than has usually been held.

| Seat of Disease. | Deaths, Registration Area: 1914 Number | Rate per 100,000 population | Per cent of aggregate |
|---|---|---|---|
| Cancer and other malignant tumors (aggregate) | 52,420 | 79.4 | 100.0 |
| Cancer of the buccal cavity (total) | 2,270 | 3.4 | 4.3 |
| Cancer of the— | | | |
| Lip | 376 | 0.6 | 0.7 |
| Tongue | 614 | 0.9 | 1.2 |
| Mouth | 230 | 0.3 | 0.4 |
| Jaw | 851 | 1.3 | 1.6 |
| Others of this class | 199 | 0.3 | 0.4 |
| Cancer of the stomach, liver (total) | 19,889 | 30.1 | 37.9 |
| Cancer of the— | | | |
| Pharynx | 58 | 0.1 | 0.1 |
| Esophagus | 605 | 0.9 | 1.2 |
| Stomach | 12,768 | 19.3 | 24.4 |
| Liver and gall bladder | 6,458 | 9.8 | 12.3 |
| Cancer of the peritoneum, intestines, rectum (total) | 6,745 | 10.2 | 12.9 |
| Cancer of the— | | | |
| Mesentery and peritoneum | 485 | 0.7 | 0.9 |
| Intestines (except rectum) | 4,055 | 6.1 | 7.7 |
| Rectum and anus | 2,171 | 3.3 | 4.1 |
| Others of this class | 34 | 0.1 | 0.1 |
| Cancer of the female genital organs (total) | 8,152 | 12.4 | 15.6 |
| Cancer of the— | | | |
| Ovary and fallopian tube | 451 | 0.7 | 0.9 |
| Uterus | 7,470 | 11.3 | 14.3 |
| Vagina and vulva | 184 | 0.3 | 0.4 |
| Others of this class | 47 | 0.1 | 0.1 |
| Cancer of the breast | 5,423 | 8.2 | 10.3 |
| Cancer of the skin | 1,957 | 3.0 | 3.7 |

| | | | |
|---|---|---|---|
| Cancer of other organs or of organs not specified (total) .......... | 7,984 | 12.1 | 15.2 |
| Cancer of the— | | | |
| Larynx .............. ...... | 341 | 0.5 | 0.7 |
| Lung and pleura ............. | 371 | 0.6 | 0.7 |
| Pancreas ..................... | 686 | 1.0 | 1.3 |
| Kidneys and suprarenals ...... | 538 | 0.8 | 1.0 |
| Prostate ...................... | 784 | 1.2 | 1.5 |
| Bladder ...................... | 1,014 | 1.5 | 1.9 |
| Brain ........................ | 141 | 0.2 | 0.3 |
| Bones (except jaw) ........... | 497 | 0.8 | 0.9 |
| Testes ....................... | 121 | 0.2 | 0.2 |
| Others of this class ........... | 3,491 | 5.3 | 6.7 |

